# Palisaded Neutrophilic and Granulomatous Dermatitis in Crohn’s Disease under Infliximab Therapy with Concomitant Herpes Zoster Infection: A Case Report

**DOI:** 10.1159/cde/abvag001

**Published:** 2026-07-28

**Authors:** Sofie Abboud, Géraldine Titeca, Léo-Paul Secco

**Affiliations:** a Department of Dermatology, Erasme Hospital, Université Libre de Bruxelles (ULB), Brussels, Belgium; b Department of Dermatology, Clinique Notre-Dame-de-Grâce, Charleroi, Belgium; c Department of Pathology, Institute of Pathology and Genetics, Charleroi, Belgium

**Keywords:** Palisaded neutrophilic and granulomatous dermatitis, Reactive granulomatous dermatitis, Inflammatory bowel disease, Crohn’s disease, Infliximab, TNF inhibitor

## Abstract

**Introduction:**

Palisaded neutrophilic and granulomatous dermatitis (PNGD) is a rare reactive dermatosis characterized clinically by symmetrical erythematous or violaceous umbilicated papules, typically involving acral sites, and histologically by a combination of neutrophilic dermatitis, interstitial granulomatous inflammation, and necrobiotic collagen. PNGD commonly arises in association with systemic conditions, especially autoimmune diseases, inflammatory arthritis, hematologic disorders, inflammatory bowel disease (IBD), infections, or medication exposure.

**Case Presentation:**

We report a 52-year-old man with longstanding Crohn’s disease treated with infliximab who developed asymptomatic erythematous papules on the nose, ear helices, and fingers following a recent herpes zoster episode and temporary interruption of infliximab therapy. Histopathology revealed an interstitial band-like infiltrate rich in neutrophils and histiocytes with basophilic necrotic collagen and leukocytoclasia, consistent with PNGD. Topical corticosteroid therapy led to complete resolution within 1 month, and no recurrence occurred after resuming infliximab over 1 year of follow-up.

**Conclusion:**

This case highlights the multifactorial nature of PNGD, in which Crohn’s disease, anti-TNF therapy, and post-zoster immune dysregulation may act as synergistic triggers. Although anti-TNF agents are known to both treat and paradoxically induce PNGD, reports in IBD remain uncommon. PNGD shares overlapping histoclinical features with interstitial granulomatous dermatitis and neutrophilic dermatoses, supporting the concept of a spectrum of reactive granulomatous dermatitis. Recognition of PNGD is essential, as it may serve as a marker of underlying systemic disease or immune dysregulation. Management focuses on treating associated conditions, with topical or systemic anti-inflammatory therapies used as adjuncts. Further studies on immunopathogenesis may refine the classification of these overlapping entities.

## Introduction

Palisaded neutrophilic and granulomatous dermatitis (PNGD) is a rare skin condition with distinct clinical and pathological characteristics. Clinically, the lesions are typically symmetrical reddish-to-bluish crusted umbilicated papules mainly located on the extremities [1–3].

Histologically, it is characterized by a combination of neutrophilic dermatitis and interstitial granulomatous dermatitis (IGD), often with necrobiotic collagen and leukocytoclasia [1–3]. This dermatological condition is frequently associated with a spectrum of systemic diseases, including connective tissue diseases, inflammatory arthritis, hematologic disorders, inflammatory bowel disease (IBD), infections, and medications [1, 2]. We report the case of a 52-year-old male with Crohn’s disease who presented multiple erythematous papules on the extremities treated by infliximab occurring after herpes zoster infection. This case report was prepared in accordance with the CAse Report (CARE) guidelines. The completed CARE checklist is provided as online supplementary material (for all online suppl. material, see https://doi.org/10.1159/cde/abvag001).

## Case Report

A 52-year-old man presented with non-pruriginous, painless erythematous papules slightly scaly symmetrically distributed on the tip of the nose, the ear lobes/helix, and the fingers over 7 days (Fig. 1). A month before, the patient reported a thoracic herpes zoster infection for which he had taken 5 days of acyclovir. The patient had a history of ano-perineal Crohn’s disease, diagnosed in 2008, complicated by an anal fistula. He has been treated with infliximab (5 mg/kg/6 weeks) since 2020. He skipped this month’s infliximab infusion due to the herpes zoster infection. The patient had no systemic symptoms except chronic diarrhea, which was already known. No blood tests were performed, given the patient’s stable condition and the absence of systemic symptoms beyond his known chronic gastrointestinal disease. The differential diagnosis mentioned was Bazex syndrome or granulomatous disease. Skin biopsy of the right helix showed a slightly ortho-keratotic and spongiotic epidermis. The main characteristic was an interstitial band-like inflammatory infiltrate occupying the dermis’s upper and middle parts. This infiltrate was rich in neutrophils and histiocytes. Basophilic necrotic collagenous tissue and leukocytoclasia were observed. No sign of vasculitis was present. The hypodermis was unremarkable (Fig. 1, 2). The clinicopathological correlation enabled the diagnosis of PNGD. Following the biopsy, the patient was treated with mometasone furoate 0.1% cream twice a day for 10 days AND then once daily for ten additional days, leading to complete resolution at the end of the 20-day treatment course. One month after treatment completion, all lesions remained absent on follow-up examination, confirming durable resolution. Subsequently, infliximab was restarted. After 1 year of follow-up, no new lesions appeared.

**Fig. 1.**
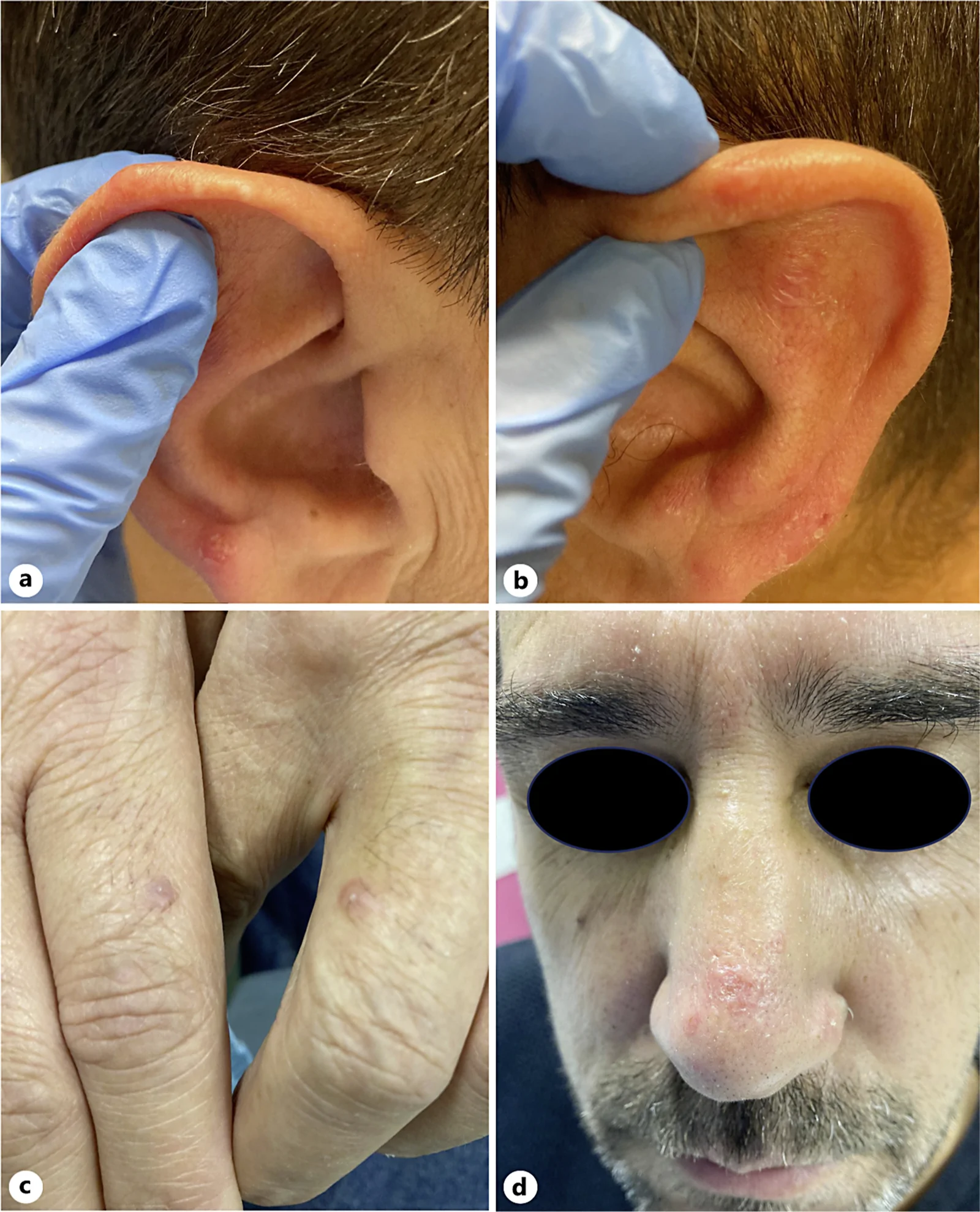
Clinical presentation of palisaded neutrophilic granulomatous dermatitis (PNGD).: **a** Right ear demonstrating small scaly erythematous infiltrated papules along the helix and inferior auricular margin. **b** Left ear with erythematous scaly papular infiltrated lesions affecting the helix and inferior auricular region. **c** Dorsal aspect of both hands showing multiple erythematous papules with central umbilication over the finger joints. **d** Nasal tip and dorsum with multiple erythematous slightly scaly papules.

**Fig. 2.**
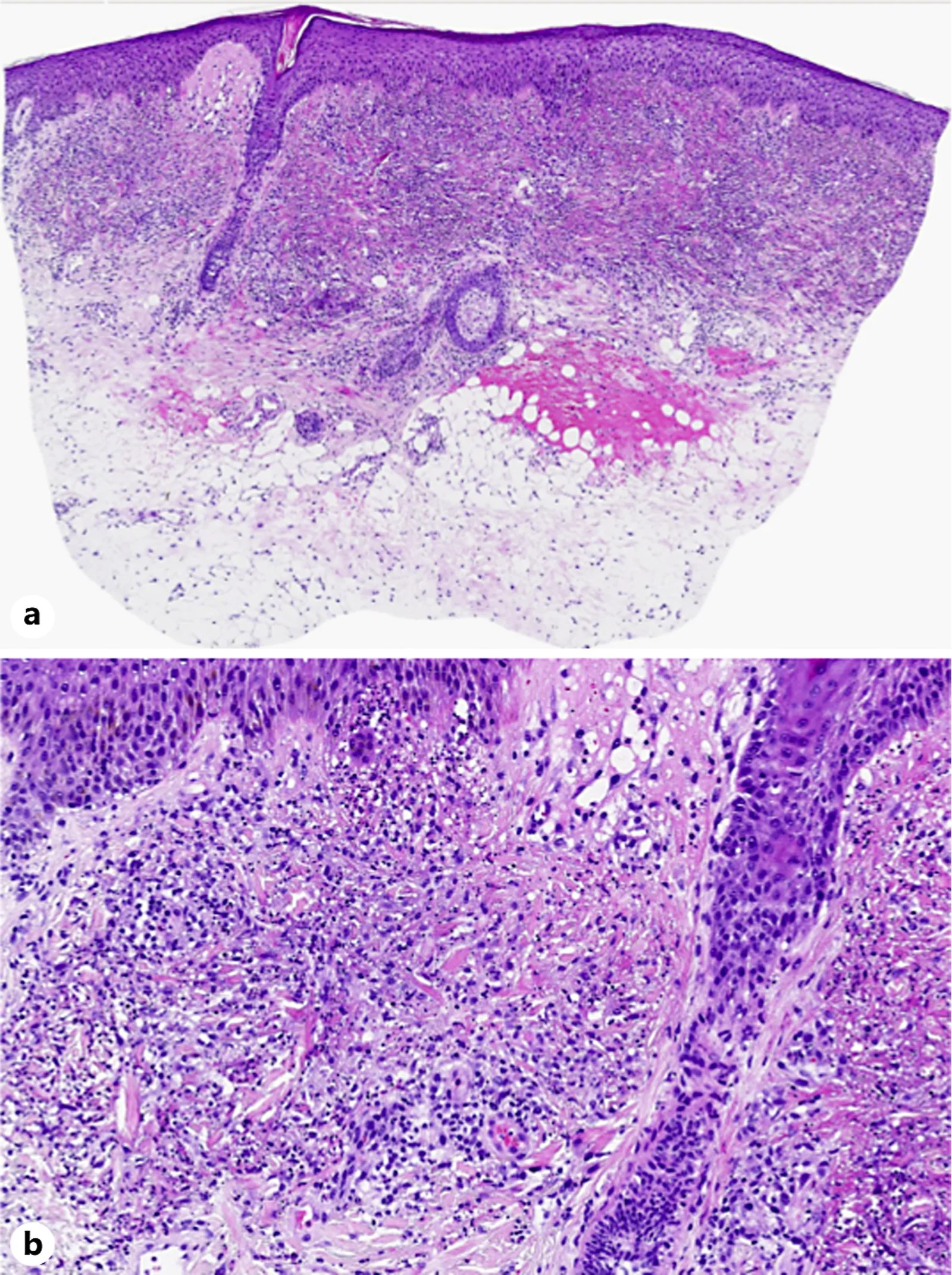
Histopathological images of the skin biopsy showing an interstitial, band-like inflammatory infiltrate composed of neutrophils and histiocytes, with areas of basophilic necrotic collagen and leukocytoclasia, consistent with palisaded neutrophilic granulomatous dermatitis (PNGD). **a** Hematoxylin and eosin (H&E) staining at low magnification (×25). **b** H&E staining at higher magnification (×100), highlighting the neutrophilic infiltrate, leukocytoclasia, and degenerated collagen.

## Discussion

PNGD is a distinct clinicopathological entity with several triggering factors involved, but no clear pathophysiological background has been identified to date. Wilmoth and Perniciaro [4] indicated that most patients with PNGD have an underlying immunoreactive condition, suggesting that PNGD is not just a symptom but a marker of systemic illness.

PNGD should be considered a reactive clinicopathological pattern rather than an isolated cutaneous disease, as it may occur in association with a wide spectrum of systemic disorders. Reported associations include autoimmune connective tissue diseases such as rheumatoid arthritis and systemic lupus erythematosus, granulomatous diseases including granulomatosis with polyangiitis, infections, IBD, medications, and hematologic malignancies [1, 2, 4–7]. Of note, myelodysplastic syndromes and chronic myelomonocytic leukemia have been increasingly recognized in association with PNGD, particularly in patients harboring SRSF2 and ASXL1 mutations [6]. In some patients, cutaneous lesions may precede the diagnosis of the underlying systemic condition and therefore represent the first clinical manifestation of systemic disease. Consequently, the diagnosis of PNGD should prompt careful clinical assessment and appropriate laboratory investigations to exclude associated autoimmune, infectious, granulomatous, or hematologic disorders. In other words, the presence of PNGD could indicate the presence of a systemic disease, serving as a diagnostic clue for clinicians [1, 2, 4–7].

Few patients with IBD have been reported with PNGD lesions, the majority of whom had ulcerative colitis [2]. One case of PNGD has been described in Crohn’s disease treated with infliximab, with a completely different clinical presentation (fever, Sweet-like cutaneous lesions, treated with oral steroids) [8].

IBD, particularly Crohn’s disease, is frequently associated with inflammatory dermatoses, including PNGD [1, 9]. These cutaneous manifestations often reflect systemic inflammatory activity. Immune complex deposition and subsequent neutrophilic activation are thought to trigger the granulomatous reaction seen in PNGD. Clinical observations suggest that cutaneous granulomas can mimic the pathological patterns found in the digestive tract, highlighting a shared pathogenic overlap between these conditions [1, 9].

Medication-induced PNGD has mainly been described in patients treated for underlying diseases that are themselves associated with PNGD, such as rheumatoid arthritis, particularly in the context of tumor necrosis factor inhibitor therapy [7]. Drug-related cases accounted for 24.1% of PNGD instances, often appearing after the prolonged use of specific medications, with TNF inhibitors (34.6%) being the most frequently implicated [1]. Other biologic agents have been reported such as diuretics, antiepileptic drugs, gout suppressant, antiviral agents, strontium, and statins [1].

TNF inhibitors are paradoxically linked to the onset of PNGD. This association highlights the dual-edged nature of anti-TNF therapy. While these biologics modulate the inflammatory cascade, they may induce or unmask hypersensitivity reactions, including granulomatous dermatitis. The exact mechanism remains elusive but is thought to involve a shift in immune complex clearance and altered neutrophilic responses [10]. Understanding these mechanisms is critical for tailoring personalized treatment regimens and underscores the importance of balancing benefits against potential dermatologic risks in patients undergoing TNF blockade [1, 10]. These observations highlight the importance of carefully monitoring cutaneous manifestations during TNF inhibitor therapy, enabling timely differentiation between treatment-related side effects and disease progression.

Several studies have reported patients receiving TNF antagonists (including infliximab) who developed PNGD; in several of these cases, the lesions resolved after discontinuation of the TNF antagonist [6, 8]. Unlike many of these previously reported cases, which were frequently associated with systemic inflammatory symptoms, fever, or extensive Sweet syndrome-like eruptions, our patient presented with localized, symmetrical acral, and facial papules without systemic manifestations. We acknowledge, however, that it is not possible to determine with certainty whether the development of PNGD in our patient was primarily triggered by the temporary interruption of infliximab, by the herpes zoster infection itself, or by both factors. The coexistence of these two events precludes any definitive conclusion regarding the predominant mechanism, and our case may therefore share a similar pathogenesis with other reported cases of PNGD following TNF inhibitor withdrawal [7]. Another distinctive feature was the favorable evolution despite reintroduction of infliximab, with no recurrence during 1 year of follow-up. This observation may support the hypothesis that transient immune dysregulation related to viral infection and interruption of TNF blockade contributed to the development of PNGD in this patient rather than infliximab toxicity itself.

Herpes zoster infections, while predominantly known for their neurocutaneous sequelae, are also implicated in reactive granulomatous processes. Post-zoster dermal changes may stimulate immune responses, leading to collagen degeneration and neutrophilic infiltration characteristic of PNGD. The interplay between viral-induced immunopathology and predisposing systemic factors warrants further investigation. Recognizing this association expands the differential diagnosis for PNGD-like lesions following viral reactivation [1, 10].

While approximately 20% of PNGD cases can resolve spontaneously, the key to PNGD treatment lies in identifying and addressing the underlying systemic disease [1, 5]. Standard therapies primarily aim at managing systemic conditions rather than PNGD [10]. Dermocorticoids, intralesional corticosteroids, dapsone, and systemic corticosteroids may be given to the patient [2, 10]. In some cases, PNGD may be a self-limited disease [2].

The differential diagnosis of PNGD includes several granulomatous and neutrophilic dermatoses with overlapping clinical and histopathological features. Clinically, PNGD may mimic granuloma annulare, rheumatoid nodules, Sweet syndrome, leukocytoclastic vasculitis, erythema elevatum diutinum, sarcoidosis, or perforating dermatoses. Histopathologically, distinguishing from IGD may be particularly challenging, as both entities fall within the spectrum of reactive granulomatous dermatitis. PNGD, IGD, and neutrophilic dermatosis share overlapping clinical and histological features, complicating diagnosis (Table 1) [3–5, 9–11]. PNGD is distinguished by palisaded histiocytes surrounding necrobiotic collagen with leukocytoclasia [1–3]. In contrast, IGD exhibits interstitial histiocytic infiltration without vasculitis but includes the characteristic floating sign of histiocytes rimming collagen [3–5, 9–11]. Neutrophilic dermatoses, like Sweet’s syndrome, primarily involve diffuse neutrophilic infiltration without granuloma formation, sometimes with leukocytoclasia or vasculitis [3–5, 9–11]. These entities reflect a spectrum of reactive granulomatous dermatitis [3–5, 9–11]. This convergence necessitates careful clinicopathological correlation to guide management and underscores the need for standardized diagnostic frameworks.

**Table 1.** Overlap of clinical and histopathological features, associated conditions, and treatment approaches between neutrophilic dermatitis, IGD, and PNGD

​	Neutrophilic dermatitis	IGD	PNGD
Histology	Neutrophils Leukocytoclasia	Histiocytes Fibrotic bundles	Neutrophils Histiocytes Necrobiotic collagen Leukocytoclasia
Clinical presentation	Polymorphous (erythematous papules, pustules, nodules, or plaques) Sometimes systemic symptoms	Erythematous to violaceous plaques Linear or cord-like Asymptomatic or pruritic Rare systemic symptoms	Umbilicated papules or nodules May be associated with systemic symptoms
Localization	Proximal and distal areas	Proximal areas	Distal areas
Associated conditions	Inflammatory disease Autoimmune disease Infection Malignancy	Autoimmune diseases (mainly rheumatoid arthritis)	Autoimmune diseases Inflammatory diseases
Treatment approaches	Treatment of the underlying causes Systemic corticosteroids Dapsone Colchicine	Treatment of the underlying causes Systemic or topical corticosteroids Antimalarials	Treatment of the underlying causes Systemic or topical corticosteroids Non-steroidal anti-inflammatory drugs Dapsone Colchicine Oral tacrolimus TNF inhibitors

## Conclusion

We report a case of PNGD arising in the context of Crohn’s disease, temporary interruption of infliximab, and concomitant herpes zoster infection – a combination of triggers not previously described. This case underscores the multifactorial nature of PNGD, in which systemic inflammatory disease, disruption of anti-TNF therapy, and viral-induced immune dysregulation may act synergistically. Whether the herpes zoster infection, the infliximab interruption, or their combination was the predominant trigger remains uncertain. The favorable outcome after resumption of infliximab, with no recurrence over 1 year, supports continued biologic therapy in well-selected patients following resolution of PNGD. PNGD shares histoclinical overlap with IGD and neutrophilic dermatoses, reinforcing the concept of a reactive granulomatous dermatitis spectrum. Recognition of PNGD as a potential marker of underlying immune dysregulation is essential, and further studies on its immunopathogenesis may refine the classification and management of these overlapping entities. Further research on the immunopathogenesis of these different clinicopathological entities could help better classify and manage them.

## Statement of Ethics

Ethical approval is not required for this study in accordance with local or national guidelines. Written informed consent was obtained from the patient for publication of the details of their medical case and any accompanying images. The CARE Checklist for this case report is available as online supplementary material.

## Conflict of Interest Statement

The authors have no conflicts of interest to declare.

## Funding Sources

This article has no funding source.

## Author Contributions

Sofie Abboud: conceptualization, writing – original draft, review, and editing, and visualization. Géraldine Titeca: review and editing. Léo-Paul Secco: review and editing. All authors approved the final manuscript.

## Data Availability

The data that support the findings of this study are not publicly available due to privacy considerations but are available from the corresponding author upon reasonable request.
